# DeepLION2: deep multi-instance contrastive learning framework enhancing the prediction of cancer-associated T cell receptors by attention strategy on motifs

**DOI:** 10.3389/fimmu.2024.1345586

**Published:** 2024-03-07

**Authors:** Xinyang Qian, Guang Yang, Fan Li, Xuanping Zhang, Xiaoyan Zhu, Xin Lai, Xiao Xiao, Tao Wang, Jiayin Wang

**Affiliations:** ^1^ School of Computer Science and Technology, Xi’an Jiaotong University, Xi’an, China; ^2^ Shaanxi Engineering Research Center of Medical and Health Big Data, Xi’an Jiaotong University, Xi’an, China; ^3^ Department of Clinical Oncology, The Second Affiliated Hospital of Air Force Medical University, Xi’an, China; ^4^ Genomics Institute, Geneplus-Shenzhen, Shenzhen, China; ^5^ Department of Thoracic Surgery, The Second Affiliated Hospital of Air Force Medical University, Xi’an, China

**Keywords:** T cell receptor, TCR repertoire data analysis, cancer-associated TCR, machine learning approach, deep learning framework, multi-instance learning, sparse self-attention, contrastive learning

## Abstract

**Introduction:**

T cell receptor (TCR) repertoires provide valuable insights into complex human diseases, including cancers. Recent advancements in immune sequencing technology have significantly improved our understanding of TCR repertoire. Some computational methods have been devised to identify cancer-associated TCRs and enable cancer detection using TCR sequencing data. However, the existing methods are often limited by their inadequate consideration of the correlations among TCRs within a repertoire, hindering the identification of crucial TCRs. Additionally, the sparsity of cancer-associated TCR distribution presents a challenge in accurate prediction.

**Methods:**

To address these issues, we presented DeepLION2, an innovative deep multi-instance contrastive learning framework specifically designed to enhance cancer-associated TCR prediction. DeepLION2 leveraged content-based sparse self-attention, focusing on the top *k* related TCRs for each TCR, to effectively model inter-TCR correlations. Furthermore, it adopted a contrastive learning strategy for bootstrapping parameter updates of the attention matrix, preventing the model from fixating on non-cancer-associated TCRs.

**Results:**

Extensive experimentation on diverse patient cohorts, encompassing over ten cancer types, demonstrated that DeepLION2 significantly outperformed current state-of-the-art methods in terms of accuracy, sensitivity, specificity, Matthews correlation coefficient, and area under the curve (AUC). Notably, DeepLION2 achieved impressive AUC values of 0.933, 0.880, and 0.763 on thyroid, lung, and gastrointestinal cancer cohorts, respectively. Furthermore, it effectively identified cancer-associated TCRs along with their key motifs, highlighting the amino acids that play a crucial role in TCR-peptide binding.

**Conclusion:**

These compelling results underscore DeepLION2's potential for enhancing cancer detection and facilitating personalized cancer immunotherapy. DeepLION2 is publicly available on GitHub, at https://github.com/Bioinformatics7181/DeepLION2, for academic use only.

## 1 Introduction

T cells are crucial elements in human immune system, capable of recognizing and responding to various antigens, including tumors, through their T cell receptors (TCRs) (1–3). In cancers, specific TCRs with distinct characteristics emerge in patients’ T cell repertoire, referred to cancer-associated TCRs (caTCRs) (4). These caTCRs possess unique adaptations to interact with tumor-related antigens, contributing to the immune response against cancer. They also exhibit shared biochemical signatures among caTCRs targeting the same cancer type or subtype, holding promise for cancer detection and treatment (5–8). Although the precise biochemical properties distinguishing caTCRs are still under exploration, advancements in the Adaptive Immune Receptor Repertoire sequencing (AIRR-seq) have revolutionized our understanding of TCR repertoires at both individual and population levels, generating vast sequencing data (9). Consequently, computational frameworks have been developed to predict caTCRs, some to differentiate cancer-associated repertoires from non-cancer ones (10–18). By leveraging the data obtained through AIRR-seq, these computational frameworks play a crucial role in early cancer screening and the prediction of cancer immune responses and immunotherapy effectiveness (15, 19). Moreover, they contribute to detecting molecular residual diseases and interpreting tumor mutation burdens, serving as pivotal biomarkers for assessing a patient’s prognostic status (20–22).

Predicting caTCRs from AIRR-seq data has been defined as a multi-instance learning (MIL) task, with individual TCRs as ‘instances’ and the entire repertoire as the ‘bag’ (17, 18, 23). Current computational methods predominantly focus on the complementarity determining region 3 (CDR3) of the TCRβ chain, involving two crucial components: CDR3 sequence feature extraction and the application of MIL techniques. Regarding sequence feature extraction, traditional methods, based on similarity comparisons of entire sequences, faced challenges in pinpointing specific amino acid residues, or ‘motifs,’ crucial for antigen recognition (11, 13). To address this limitation, some researchers preprocessed sequences into fixed-length overlapping fragments (10, 12, 14). However, motif lengths remained variable, constraining their performance (14). DeepLION first designed the model that enables to accommodate motifs of various lengths, surpassing existing methods in feature extraction (17). In the context of applying MIL techniques, early methods primarily considered the most significant CDR3 sequence, neglecting other valuable sequences (10–15). To address this issue, DeepTCR employed a multi-head attention mechanism, while DeepLION used a linear classifier, assigning appropriate weights to CDR3 sequences in the repertoire (16, 17). To further tackle the issue of a small fraction of caTCRs within the repertoire, MINN_SA applied a sparsity constraint to the linear classifier’s output, focusing attention on the caTCRs within the repertoire, and achieved superior performance compared to popular MIL methods (15, 18).

Unfortunately, in the application of MIL techniques, there are still two key issues that prevent accurate predictions from the existing methods: their inadequate consideration of the correlations among the TCRs within a repertoire, and the sparsity of caTCR distribution. On one hand, TCRs with similar or even identical CDR3 sequences can recognize different antigens based on their distinct structural characteristics (24). Consequently, those sequence-based methods are susceptible to misclassification in such cases, including mislabeling non-cancer TCRs with similar or identical sequences to caTCRs as caTCRs, resulting in false positives. Fortunately, utilizing the context of TCRs, specifically calculating the correlations between TCRs, enhances the inference of TCR antigen-binding specificity and enables an accurate caTCR prediction (25). While TransMIL effectively integrated the self-attention mechanism within its MIL component to capture inter-instance correlations, showing impressive performance in whole slide image classification (26), dedicated methods for caTCR prediction remain to be developed. On the other hand, tumor-infiltrating lymphocyte repertoires often contain over 80% of TCRs lacking tumor reactivity, indicating the sparse distribution of caTCRs (15, 27). In such cases, the self-attention mechanism calculates a group of attention scores for a TCR compared to all others, which may inadvertently allocate excessive attention to unrelated TCRs, and further generate erroneous predictions. In addition, insufficient samples from patients with the same cancer type may impede the model’s ability to focus on the sparse caTCRs, limiting the prediction performance of the self-attention mechanism (25, 26).

In summary, the current computational methods for caTCR prediction are constrained by their limited consideration of the correlations among TCRs in the repertoire and the sparsity of caTCR distribution. To address these issues, we proposed a novel MIL method called DeepLION2, which incorporated sparse self-attention and contrastive learning, to enhance the prediction of caTCRs using TCR sequencing (TCR-seq) data. It met the requirement to consider TCR correlations and to identify sparse caTCRs within the repertoire by utilizing a content-based sparse attention mechanism. This mechanism focused only on the *k* most relevant TCRs for each TCR, avoiding unnecessary attention on unrelated TCRs. Additionally, we integrated a self-contrastive learning strategy into model training to enhance the attention matrix by focusing on sparse caTCRs and thereby improving caTCR prediction. In our nested cross-validation evaluation, DeepLION2 outperformed the state-of-the-art methods in caTCR prediction and repertoire classification and achieved impressive area under the receiver operating characteristic (ROC) curve (AUC) values of 0.933, 0.880, and 0.763 on raw TCR-seq data of thyroid cancer (THCA), lung cancer (LUCA), and gastrointestinal cancer (GICA) patient cohorts, respectively. Moreover, it could effectively identify caTCRs along with their key motifs, which are essential for TCR-peptide binding. These results highlight its potential to advance cancer research and facilitate personalized cancer immunotherapy.

## 2 Materials and methods

**Figure 1:**
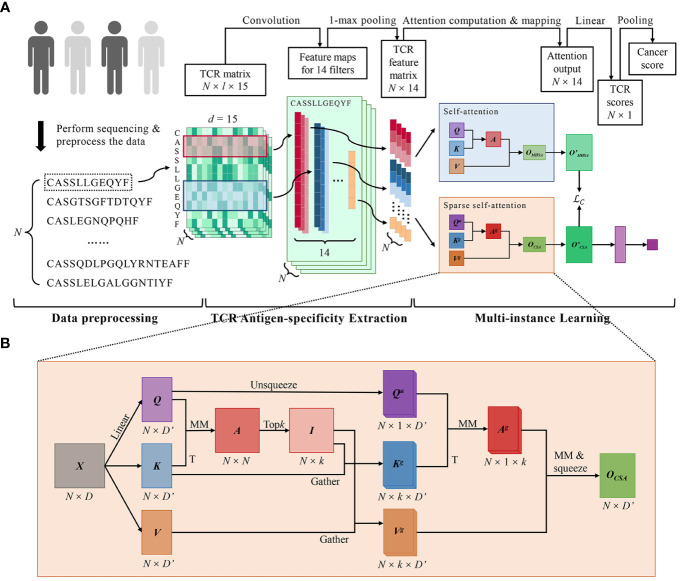
DeepLION2 for accurate prediction of cancer-associated TCRs. **(A)** The workflow of DeepLION2 contained three parts: data preprocessing, TCR antigen-specificity extraction, and multi-instance learning. In the data preprocessing, raw sequences of length *l* were embedded into the TCR matrix with dimension *l* × 15 after sequence filtering. Then the antigen-specificity of each TCR was extracted by a convolutional network and the corresponding feature was generated. In the last part, DeepLION2 used a content-based sparse self-attention to capture the correlations between each TCR and its top *k* related TCRs. Moreover, it also performed self-attention calculation for self-contrastive learning, where the outputs of sparse self-attention and self-attention were compared to improve attention learning. Finally, the attention output was linearly mapped and pooled to generate the cancer score for the repertoire. **(B)** The computational details of content-based sparse self-attention used in DeepLION2. First, the TCR-to-TCR affinity graph A was calculated with *Q* and *K*, measuring the correlation between TCRs. Then the index matrix I was derived with the row-wise extraction operation, which recorded the *k* indices of the related TCRs for each TCR. In the subsequent computation of self-attention, the *k* most relevant TCRs for each TCR were selectively considered, thereby mitigating the impact of less relevant TCRs. Finally, the output $O_{CSA}$ was obtained after the computation of self-attention. MM, matrix multiplication; T, transposition.

DeepLION2 set up a three-component workflow, comprising data preprocessing, TCR antigen-specificity extraction, and MIL, which closely resembled the workflow of DeepLION (17) (**Figure 1A**). While the initial two parts of DeepLION2 employed the same methodology as DeepLION for data preprocessing and TCR antigen-specificity extraction, the main improvement was observed in the MIL component. In the third part, DeepLION2 introduced a content-based sparse self-attention mechanism in conjunction with contrastive learning to effectively aggregate TCR features and embed the repertoire. It performed both self-attention and sparse self-attention computations and compared the results to optimize attention learning. By considering the relationships among TCRs within the repertoire and the sparsity of caTCRs, it significantly enhanced the aggregation process, enabling accurate prediction of whether the TCR repertoire was cancerous or non-cancerous.

### 2.1 Data preprocessing

To effectively utilize TCR-seq data for caTCR prediction and repertoire classification, preprocessing steps are necessary, involving sequence filtering and embedding. Considering that existing studies on TCRs predominantly focused on the β chain, we only kept the β chain CDR3 sequences as the input of DeepLION2. During sequence filtering, low-quality CDR3 sequences and those unrelated to cancer were removed. As described in the previous studies (15, 17), the following types of sequences were removed: I. sequences with inadequate length (< 10) or excessive length (> 24), II. sequences featuring special characters (X, +, *, etc.), III. incomplete sequences, not commencing with cysteine (C) or culminating with phenylalanine (F), IV. sequences with an unresolved variable gene locus, and V. sequences appearing in the reference dataset from Xu’s study, frequently observed in both healthy individuals and cancer patients. From the remaining sequences, the *N* sequences with the highest abundance were selected. In the sequence embedding process, sequences were encoded into numerical matrices that effectively contained the antigen-binding specificity of the CDR3 for downstream analysis. A sequence of length *l* could be encoded into an *l* × *d* TCR matrix using a 20 × *d* feature matrix, where each of the 20 amino acids was represented by a feature vector of dimension *d*. Among popular feature matrices (15, 28, 29), given that the Beshnova matrix contains more biochemical information and has demonstrated good performance in methods like DeepLION and DeepCAT, our method also adopted it for encoding sequences of length *l* into an *l* × 15 TCR matrix.

### 2.2 TCR antigen-specificity extraction considering the cancer-associated motifs of different lengths

Properly extracting TCR antigen-specificity is essential for identifying caTCRs, and various computational methods have been used for this purpose. Compared with other methods, DeepLION introduced a convolution network with convolutional filters of different sizes to consider the key motifs of different lengths in TCRs, resulting in improved performance (17). As a result, DeepLION2 adopted a similar network architecture to DeepLION to extract TCR antigen-specificity. It consisted of 14 convolutional filters with different sizes that performed convolution operations on the TCR matrix, generating corresponding convolution mappings. The 1-max pooling function was then applied to reduce each mapping dimension to 1. By concatenating these mapping results, a feature with a dimension of 1 × 14 representing the TCR antigen-specificity was obtained for each TCR. Finally, this process resulted in an *N* × 14 matrix for each TCR repertoire.

### 2.3 Multi-instance learning properly modeling the relationships among TCRs

#### 2.3.1 Self-attention for calculating the correlation scores between TCRs

Considering the relationships among TCRs within the repertoire can better estimate the antigen-binding specificity of TCRs with similar/identical CDR3 sequences. Self-attention-based models have proven to be quite effective in processing sequence data and calculating relationship scores between instances in MIL. Similar to these models, the self-attention mechanism in DeepLION2 was formally defined as Equations (1, 2):


(1) $$\begin{array}{c} Q=XW^{q}+b^{q},K=XW^{k}+b^{k},V=XW^{v}+b^{v},\; \end{array}$$

(2) $$\begin{array}{c} O_{SA}=\text{Attention}\left(Q,K,V\right)=\text{softmax}\left(\frac{QK^{\text{T}}}{\sqrt{D^{\prime }}}\right)V, \end{array}$$

where $X\in {\mathbb{R}}^{N\times D}$ was an input with *N* instances, whose dimension was *D* (*D* = 14 in DeepLION2 due to the TCR feature matrix), $Q\in {\mathbb{R}}^{N_{q}\times D^{\prime }}$ , $\;K\in {\mathbb{R}}^{N_{kv}\times D^{\prime }}$ and $V\in {\mathbb{R}}^{N_{kv}\times D^{\prime }}$ were query, key and value matrices obtained by linear transformation of *X*, where D' was the dimension of the instance after transformation, and softmax(·) was the activation function to normalize the results and get the final output $O_{SA}\in {\mathbb{R}}^{N\times D^{\prime }}$ . The output of $\text{softmax}\left({\mathrm{QK}}^{\text{T}}/\sqrt{D'}\right)\in {\mathbb{R}}^{N\times N}$ could be seen as the attention score matrix computed, containing the relationship scores between the corresponding instances. To avoid weight concentration and gradient vanishing, the scalar factor $\sqrt{D'}$ was introduced (25).

To improve the self-attention mechanism’s performance, a preferred method is multi-head self-attention, formally defined as Equations (3–5):


(3) $$\begin{array}{c} Q=\left\{Q_{1},Q_{2},\ldots ,Q_{h}\right\},K=\left\{K_{1},K_{2},\ldots ,K_{h}\right\},V=\left\{V_{1},V_{2},\ldots ,V_{h}\right\}, \end{array}$$

(4) $$\begin{array}{c} Head_{i}=\text{Attention}\left(Q_{i},K_{i},V_{i}\right), \end{array}$$

(5) $$\begin{array}{c} O_{\mathrm{MHSA}}=\text{MHSA}\left(X\right)=\text{Concat}\left(Head_{1},Head_{2},\ldots ,Head_{h}\right). \end{array}$$

In the multi-head self-attention computation process, *Q*, *K* and *V* were divided into *h* equal parts, where $Q_{i}$ , $K_{i}$ and $V_{i}\in {\mathbb{R}}^{N\times \left(D^{\prime }/h\right)}$ were used to computed the *i*th attention head $Head_{i}\in {\mathbb{R}}^{N\times \left(D^{\prime }/h\right)}$ . And then the outputs of *h* heads were concatenated as the final attention output $\;O_{MHSA}\in {\mathbb{R}}^{N\times D^{\prime }}$ . To facilitate the stacking of self-attention blocks, the output $O_{MHSA}$ was linearly transformed to the output OMHSA'∈ℝN×D , whose dimension was the same as the input *X* as Equation (6):


(6) $$\begin{array}{c} O_{MHSA}^{'}=O_{MHSA}W^{A}+b^{A}. \end{array}$$

In the context of caTCR prediction, the input *X* of the self-attention was the *N* × 14 TCR repertoire feature matrix, where each row represented a TCR and *N* represented the total number of TCRs. This mechanism calculated correlation scores between TCRs, enabling the accurate extraction of cancer-associated biochemical features and precise identification of caTCRs within the repertoire.

#### 2.3.2 Content-based sparse self-attention prioritizing top *k* related TCRs for each TCR

Due to the sparsity of caTCRs, calculating relationship scores between all TCRs may inadvertently shift the focus toward non-cancer-associated TCRs, thereby potentially reducing caTCR prediction performance. To tackle this, the preferred solution is sparse self-attention, which falls into two categories: position-based and content-based approaches (30, 31). Position-based attention restricts the attention matrix based on predefined position-related patterns, but the distribution pattern of caTCRs in the repertoire remains unknown. As a result, DeepLION2 incorporated the content-based sparse attention to only consider the top *k* related TCRs for each TCR in the repertoire (**Figure 1B**). The formal definition was as Equations (7–11):


(7) $$\begin{array}{c} A=QK^{\text{T}}, \end{array}$$

(8) $$\begin{array}{c} I=\text{topkIndex}\left(A\right), \end{array}$$

(9) $$\begin{array}{c} Q^{u}=\text{unsqueeze}\left(Q\right),\;K^{g}=\text{gather}\left(K,I\right),V^{g}=\text{gather}\left(V,I\right), \end{array}$$

(10) $$\begin{array}{c} O_{CSA}=\text{CSA}\left(X\right)=\text{squeeze}\left(\text{Attention}\left(Q^{u},K^{g},V^{g}\right)\right), \end{array}$$

(11) $$\begin{array}{c} O_{CSA}^{'}=O_{CSA}W^{A}+b^{A}. \end{array}$$

First, we derived the TCR-to-TCR affinity graph $A\in {\mathbb{R}}^{N\times N}$ via matrix multiplication between *Q* and transposed *K*, where measured the correlation of each TCR with other TCRs. And then we pruned the affinity graph A by retaining only the first *k* connections of each TCR based on the values of the elements in A (i.e., the correlation between TCRs). The index matrix $I\in {\mathbb{R}}^{N\times k}$ was derived with the row-wise extraction operation, which recorded the *k* indices of the related TCRs for each TCR. Specifically, the *i*th row of *I* included *k* indices of the most relevant TCRs for the *i*th TCR. The gathered key and value matrices $K^{g}$ and $V^{g}\in {\mathbb{R}}^{N\times k\times D^{\prime }}$ , which contained the top *k* TCR vectors for each TCR, were obtained by gathering *K* and *V* with *I* (i.e., extracting the corresponding elements according to the indices in *I*). For facilitating the following matrix multiplication computations, the unsqueezed query matrix $Q^{u}\in {\mathbb{R}}^{N\times 1\times D^{\prime }}$ was obtained by ascending the dimension of *Q*. Finally, the self-attention was applied on the *Q^u^*, *K^g^* and *V^g^*, and the output $O_{CSA}\in {\mathbb{R}}^{N\times D^{\prime }}$ was obtained after the squeeze operation. Consistent with the multi-head self-attention, we obtained the final output OCSA'∈ℝN×D after the linear transformation. In conclusion, by precomputing TCR correlations before applying traditional self-attention, DeepLION2 selectively considered the *k* most relevant TCRs for each TCR, thereby mitigating the impact of less relevant TCRs.

#### 2.3.3 Self-contrastive learning for robust attention learning

Content-based sparse self-attention has shown excellent performance on classification tasks when sufficient training data (> one thousand samples) is available (31). However, obtaining TCR-seq data from a sufficient number of patients with the same cancer type is challenging, and if there isn’t enough training data, the model struggles to focus on the sparse caTCRs in the repertoire. To address this challenge, DeepLION2 incorporated self-contrastive learning in its MIL component. We first assumed that each TCR within a repertoire exclusively relates to others recognizing the same antigen, signifying an attention score of 0 with unrelated TCRs. Based on this assumption, we inferred that the output, despite lacking specific constraints in self-attention calculation, would be identical to that derived from sparse self-attention. To ensure this, we performed both self-attention and sparse self-attention calculations and compared their outputs using the mean square error loss function, in the aim to minimize the discrepancy between the two outputs during the model training. The loss function was defined as Equation (12):


(12) $$\begin{array}{c} {ℒ}_{C}=\text{MSE}\left(O_{CSA}^{'},O_{MHSA}^{'}\right)=\frac{1}{N\cdot D}\sum_{i=1}^{N}\sum_{j=1}^{D}{\left(o_{ij}-{\hat{o}}_{ij}\right)}^{2}, \end{array}$$

where $o_{ij}$ and ${\hat{o}}_{ij}\;$ were the elements of the *i*-th row and *j*-th column in the output matrix $O_{CSA}^{'}$ and $O_{MHSA}^{'}$ . In the model training process, by optimizing the loss function ${ℒ}_{\text{C}}$ , the attention score matrix of the sparse self-attention was constantly revised, where each TCR focused only on relevant TCRs and ignored other TCRs. Consequently, this strategy allowed DeepLION2 to focus more on the sparse caTCRs within a repertoire with small sample sizes.

#### 2.3.4 Decision layer for making prediction both for TCRs and repertoires

The decision layer was designed to make the final predictions for individual TCRs and the TCR repertoire. The output of the content-based sparse self-attention $O_{CSA}^{'}$ was linearly transformed to integrate the features of each TCR, as Equation (13):


(13) $$\begin{array}{c} \tilde{y}=\text{Sigmoid}(O_{CSA}^{'}W^{D}+b^{D}), \end{array}$$

where $W^{D}\in {\mathbb{R}}^{D\times 1}$ and $b^{D}\in {\mathbb{R}}^{D}$ were the weight and the bias of the linear transformation, Sigmoid(·) was the activation function to map the values to the interval (0, 1), and $\tilde{y}\in {\mathbb{R}}^{N\times 1}$ represented the prediction scores of TCRs in the repertoire. And then the average pooling was used to mapping the $\tilde{y}$ into the TCR repertoire prediction result as Equation (14):


(14) $$\begin{array}{c} \tilde{Y}=P\left(Y=1|\tilde{y}\right)=\frac{1}{N}\sum_{i=1}^{N}{\tilde{y}}_{i}, \end{array}$$

where $\text{P}\left(Y=1|\tilde{y}\right)$ denoted the probability that the TCR repertoire is associated with cancer (i.e., the probability that a patient has cancer). When $\tilde{Y}>0.5$ , the repertoire was predicted to be cancer-associated, and to be noncancerous otherwise.

The whole model DeepLION2 was end-to-end trainable, and the loss function ℒ used for model training was defined as Equations (15, 16):


(15) $$\begin{array}{c} {ℒ}_{M}=\text{CE}\left(\tilde{Y},Y\right)=-\left[Y\cdot \text{log}\left(\tilde{Y}\right)+\left(1-Y\right)\cdot \text{log}\left(1-\tilde{Y}\right)\right], \end{array}$$

(16) $$\begin{array}{c} ℒ={ℒ}_{M}+{ℒ}_{C}, \end{array}$$

where ℒ consisted of the main loss function ${ℒ}_{\text{M}}$ and the self-contrastive learning loss function ${ℒ}_{\text{C}}$ . By optimizing the log-likelihood function ${ℒ}_{\text{M}}$ , DeepLION2 learned to predict whether a repertoire is cancer-associated. Additionally, a constraint term ${ℒ}_{C}$ was optimized to enhance the learning of sparse self-attention and improve prediction performance.

The trained DeepLION2 not only predicted the cancer status of patient samples but also identified caTCRs within a repertoire through the TCR score vector $\tilde{y}$ . Each element ${\tilde{y}}_{i}$ in the vector represented the probability that the *i*th TCR in the repertoire is a caTCR. In a predicted cancerous repertoire, the probability ${\tilde{y}}_{i}$ served as a reliable indicator: the higher the probability, the stronger the likelihood that the corresponding TCR is associated with cancer. Conversely, regardless of their respective probabilities, every TCR in a predicted noncancerous repertoire is unassociated with cancer. Additionally, DeepLION2 could also identify the key motifs of caTCRs by calculating the motif scores according to the weight parameters of the trained model.

## 3 Results

To evaluate the performance of DeepLION2, we conducted experiments on diverse cohorts of patients with various cancer types. We first described in detail the experimental data, comparison models, evaluation metrics, and cross-validation approach. Then we specifically assessed the enhancement of the MIL component of DeepLION2 using preprocessed real data. Furthermore, we applied DeepLION2 to raw TCR-seq data to assess its performance when predicting the caTCRs and repertoires. Finally, we demonstrated the key TCRs with their motifs from the raw data based on the trained models.

### 3.1 Collecting data

We utilized two real datasets encompassing more than 10 cancer types for our experiments. The first dataset was obtained from The Cancer Genome Atlas (TCGA) database, comprising paired tumor and normal tissue samples from patients with ten distinct cancer types (32). These samples underwent preprocessing steps, including next-generation sequencing, TCR reconstruction techniques, and TCR encoding algorithms, as outlined in previous studies (33). Xiong et al. utilized this dataset in their review to evaluate the performance of existing MIL methods in cancer detection tasks, while Kim et al. also employed it for comparisons with other methods (18). Therefore, we employed this dataset to evaluate the MIL component of DeepLION2.

The second dataset was collected from the clinical database of Geneplus Technology Ltd. in Shenzhen (Geneplus) (34–36). It consisted of raw TCR-seq data samples, including peripheral blood mononuclear cell and tumor-infiltrating lymphocyte samples from patients with THCA, LUCA, and GICA. Additionally, non-cancer individual peripheral blood mononuclear cell samples were included as the control cohorts. The LUCA samples encompassed samples of two cancer subtypes: lung squamous cell carcinoma (LUSC) and lung adenocarcinoma (LUAD), while the GICA samples were from patients of esophageal, gastric, colorectal, hepatocellular, and pancreatic cancers. Detailed information regarding the experimental data can be found in **Table 1**.

**Table 1:** The details of the data used in the experiments.

Dataset	Disease	Disease size	Control size	Total size	Data source
TCGA	BRCA	202	202	404	(33)
	DLBC	45	45	90	(33)
	ESCA	166	166	332	(33)
	KIRC	202	202	404	(33)
	LUAD	202	202	404	(33)
	LUSC	202	202	404	(33)
	OV	202	202	404	(33)
	SKCM	202	202	404	(33)
	STAD	202	202	404	(33)
	THYM	108	108	216	(33)
Geneplus	THCA	170	260	430	(34)
	LUCA	184	260	444	(36)
	GICA	151	260	411	(35)

TCGA, The Cancer Genome Atlas; BRCA, breast invasive carcinoma; DLBC, lymphoid neoplasm diffuse large B-cell lymphoma; ESCA, esophageal carcinoma; KIRC, kidney renal clear cell carcinoma; LUAD, lung adenocarcinoma; LUSC, lung squamous cell carcinoma; OV, ovarian serous cystadenocarcinoma; SKCM, skin cutaneous melanoma; STAD, stomach adenocarcinoma; THYM, thymoma; THCA, thyroid cancer; LUCA, lung cancer; GICA, gastrointestinal cancer.

### 3.2 Comparison model settings, evaluation metrics and validation approaches

To assess the enhancements introduced by DeepLION2, we compared it with several state-of-the-art methods: DeepTCR (16), DeepLION (17), MINN_SA (18), TransMIL (26), and BiFormer (31) (**Table 2**). DeepTCR, DeepLION, and MINN_SA are embedded-space MIL methods specifically designed for TCR prediction. Due to their MIL design, DeepTCR is widely used in TCR studies, whereas DeepLION has been demonstrated to outperform earlier caTCR prediction methods, including DeepCAT (15). MINN_SA further took into account the sparsity of caTCRs and utilized sparse attention to selectively focus on the key TCRs within the repertoire while disregarding others, which has proven to perform better than popular existing MIL methods in this task. By contrast, TransMIL and BiFormer are not specific for TCR prediction. TransMIL employed the self-attention mechanism to consider inter-instance correlations and achieved significant improvement in whole slide image classification. BiFormer, a recent content-based sparse attention method in the field of computer vision, introduced bi-level routing attention and achieved higher classification accuracy than other self-attention-based methods. In order to ensure a fair comparison, we modified their network to predict TCRs by utilizing the same TCR feature extraction component as DeepLION2 and keeping only one layer as the MIL component.

**Table 2:** Summary of the comparison models.

Model	Specific for TCR prediction?	Considering correlations among instances?	Considering the sparsity of instance distribution?
DeepTCR	√	×	×
DeepLION	√	×	×
MINN_SA	√	×	√
TransMIL	×	√	×
BiFormer	×	√	√

The hyperparameters of DeepLION2 contained the number of selected TCRs in date preprocessing *N*, the dimension D' and the head number *h* of self-attention/sparse self-attention, the ratio of sparse self-attention *k_r_* (*k_r_* = *k/N*), as well as the learning rate *l_r_* and the epoch number *e* of model training. In the experiments, alignment with DeepLION, *N*, *l_r_*, and *e* were set to 100, 0.001, and 500, whereas D' , *h*, and *k_r_* were set to 10, 1, and 0.05 for low computational cost. For the comparison methods, we utilized the default hyperparameters, and DeepTCR only accepted TCRβ sequences as input.

To assess the performance of DeepLION2 and the comparison models, we employed commonly used performance metrics in machine learning and statistical analysis within the biomedical field. These metrics included accuracy (ACC), sensitivity (SEN), specificity (SPE), Matthews correlation coefficient (MCC), and AUC. ACC, SEN, SPE, and MCC could be formally defined as Equations (17–20):


(17) $$\begin{array}{c} \text{ACC}=\frac{TP+TN}{TP+TN+FP+FN}, \end{array}$$

(18) $$\begin{array}{c} \text{SEN}=\frac{TP}{TP+FN}, \end{array}$$

(19) $$\begin{array}{c} \text{SPE}=\frac{TN}{TN+FP}, \end{array}$$

(20) $$\begin{array}{c} \text{MCC}=\frac{\text{TP}\times \text{TN}-\text{FP}\times \text{FN}}{\sqrt{\left(\text{TP}+\text{FP}\right)\left(\text{TP}+\text{FN}\right)\left(\text{TN}+\text{FP}\right)\left(\text{TN}+\text{FN}\right)}},\; \end{array}$$

where *TP* is the number of correct predictions in the positive samples, whereas *FN* is the number of wrong predictions in the positive samples, and *TN* is the number of correct predictions in the negative samples, whereas *FP* is the number of wrong predictions in the negative samples. Among the metrics, MCC is a correlation coefficient that quantifies the relationship between the true class and the prediction results, ranging from -1 to 1.

*K*-fold cross-validation is a widely used validation approach for assessing model generalization. In *K*-fold cross-validation, the dataset was randomly divided into *K* equal parts, and *K* validations were performed, with each part serving as the test data while the remaining parts were used for training. This process ensures that the entire dataset is evaluated, providing an average performance estimation for the model. Unfortunately, it has been reported that *K*-fold cross-validation may yield skewed performance estimates when dealing with small sample sizes (17, 37, 38). To overcome this limitation, a refined technique called *K*-*K’*-fold nested cross-validation was introduced. This enhanced approach aims to generate robust and unbiased performance estimates, irrespective of dataset size. In each of the *K* validations within the nested cross-validation, the training data was further divided into *K’* equal parts, and *K’*-fold cross-validation was performed to select the final models. As a result, considering the small sample size of the dataset used in the experiments, a 5-4-fold nested cross-validation approach was adopted to validate the performance of the models in our experiments.

### 3.3 Sparse self-attention and contrastive learning improves multi-instance learning for prediction of cancer-associated TCRs and repertoires

**Figure 2:**
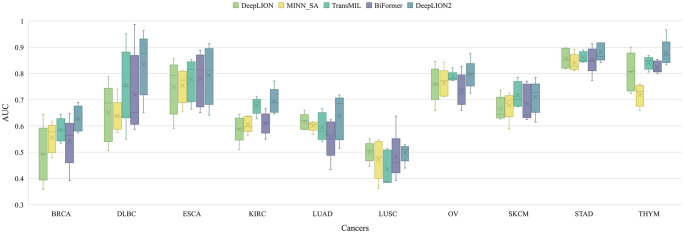
The AUC results of models on 10 cancer type samples from TCGA. BRCA, breast invasive carcinoma; DLBC, lymphoid neoplasm diffuse large B-cell lymphoma; ESCA, esophageal carcinoma; KIRC, kidney renal clear cell carcinoma; LUAD, lung adenocarcinoma; LUSC, lung squamous cell carcinoma; OV, ovarian serous cystadenocarcinoma; SKCM, skin cutaneous melanoma; STAD, stomach adenocarcinoma; THYM, thymoma; AUC, area under the receiver operating characteristic curve.

DeepLION2 concentrated on optimizing the MIL component to raise caTCR prediction accuracy. To achieve this improvement, content-based sparse self-attention was added, which helps to find important TCRs in the repertoire. Furthermore, the quality of attention-based learning outcomes was improved through the use of self-contrastive learning. To validate the effectiveness of these improvements, we compared the MIL component of DeepLION2 with those of other models using the TCGA dataset. Given that the relationship among TCRs and caTCR sparsity is not considered by either DeepTCR or DeepLION, we selected only DeepLION as a representative. For a fair comparison, we directly tested the models using 5-4-fold nested cross-validation on preprocessed samples from 10 different cancer types, without any additional processing. The validation results for all models across the ten cancer types were analyzed in terms of AUC and visualized in **Figure 2**. Additionally, the results including all metrics can be found in **Supplementary Table 1**. To facilitate comparison, the mean validation results for all metrics, across the ten cancer types, were summarized in **Table 3**. These results could provide a comprehensive overview of the performance of the models, allowing for a detailed assessment of their effectiveness in predicting caTCRs.

**Table 3:** Mean results of models across 10 cancer type samples from TCGA.

	DeepLION	MINN_SA	TransMIL	BiFormer	DeepLION2
ACC	0.631 ± 0.066	0.629 ± 0.054	0.655 ± 0.058	0.627 ± 0.067	**0.673 ± 0.057**
SEN	0.592 ± 0.112	**0.672 ± 0.117**	0.585 ± 0.130	0.580 ± 0.129	0.596 ± 0.139
SPE	0.671 ± 0.085	0.580 ± 0.118	0.722 ± 0.072	0.676 ± 0.123	**0.751 ± 0.129**
MCC	0.266 ± 0.131	0.258 ± 0.114	0.317 ± 0.113	0.264 ± 0.135	**0.367 ± 0.112**
AUC	0.669 ± 0.067	0.663 ± 0.049	0.703 ± 0.054	0.678 ± 0.077	**0.735 ± 0.066**

The maximum values of the evaluation metrics among the comparison models are shown in bold. ACC, accuracy; SEN, sensitivity; SPE, specificity; MCC, Matthews correlation coefficient; AUC, area under the receiver operating characteristic curve.

According to **Figure 2**, although DeepLION2 had slightly lower performance compared to DeepLION on LUSC samples and TransMIL on skin cutaneous melanoma samples, it generally performed better than the other four models in terms of average AUC validation results across the other eight patient cohorts. The results in **Table 3** also demonstrated that DeepLION2 achieved the highest average performance in terms of ACC, SPE, MCC, and AUC among the five models evaluated across the ten cancer types, whereas it obtained the second-highest SEN. As shown in **Table 3**, the results highlighted that DeepLION and MINN_SA, which did not consider the correlations among TCRs, exhibited lower SPEs compared to the other models. This suggested that they may be more susceptible to making incorrect predictions on negative samples and having higher false positive rates. On the other hand, TransMIL, which incorporated self-attention to capture TCR correlations, showed higher ACC, SPE, MCC, and AUC, indicating superior classification ability. While BiFormer utilized sparse self-attention to address the sparsity of caTCR distribution, its performance declined compared to TransMIL, probably because of erroneous attention learning brought on by the small sample size. In contrast, DeepLION2 leveraged self-contrastive learning to enhance sparse self-attention learning, resulting in improved predictions of caTCRs and repertoires in terms of all metrics. As a result, the MIL component of DeepLION2 excelled in effectively identifying caTCRs within the repertoire for caTCR prediction by combining sparse self-attention and contrastive learning.

It is noteworthy that the models’ performance varied among cancer types and that they underperformed in some cases, like LUSC (**Figure 2**). These variations are, in part, due to the TCR feature extraction method. The autoencoder may not have appropriately focused on the motifs when extracting features from the samples in the TCGA dataset in the previous processing (33). Consequently, poor feature extraction resulted in poor prediction performance. On the other hand, this phenomenon might have been influenced by the heterogeneity among cancer types. Simultaneously, we conjectured that, despite their similar functions, caTCRs in the repertoires of cancer types with low performances differed significantly in sequence form as a result of the structural folding of proteins. The variation in sequences of caTCRs made it difficult for computational methods to predict with accuracy.

### 3.4 DeepLION2 advances prediction of cancer-associated TCRs and repertoires based on TCR sequencing data

**Figure 3:**
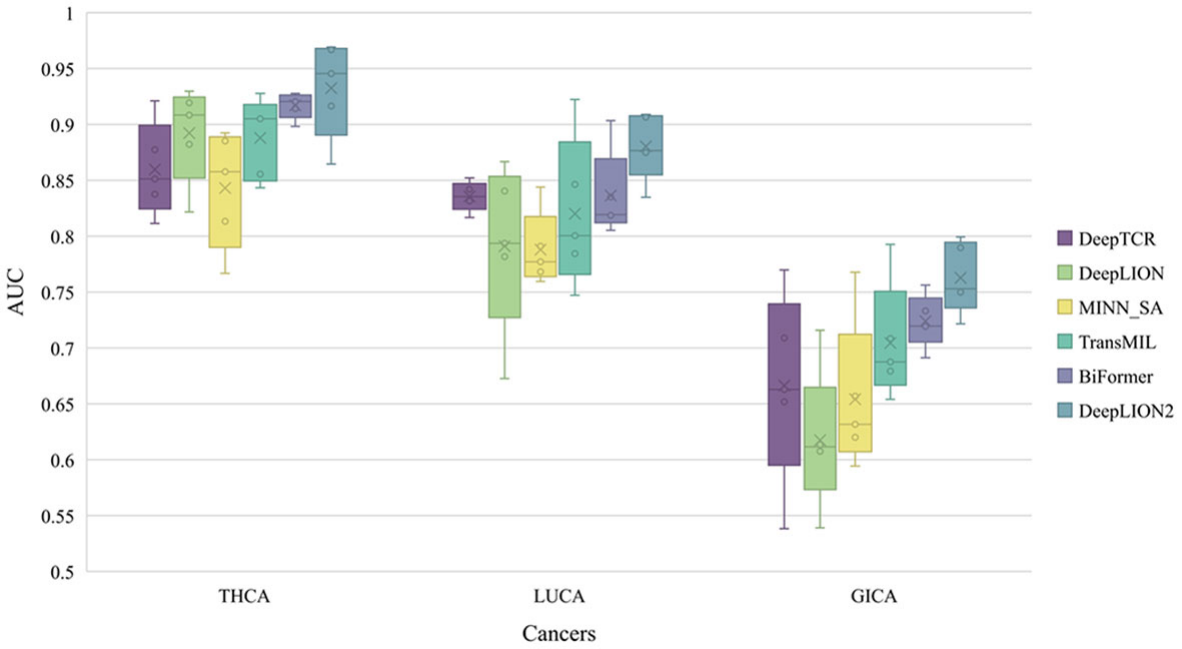
The AUC results of models on 3 cancer type samples from Geneplus. THCA, thyroid cancer; LUCA, lung cancer; GICA: gastrointestinal cancer; AUC, area under the receiver operating characteristic curve.

To thoroughly assess the models’ performance in predicting caTCRs and TCR repertoires using raw TCR-seq data, we conducted experiments on the Geneplus dataset. We employed 5-4-fold nested cross-validation to test all the models on the THCA, LUCA, and GICA patient cohorts from the Geneplus dataset. The AUC results of the models on the three cohorts are shown in **Figure 3**, and the validation results of all metrics are shown in **Table 4**.

**Table 4:** The validation results of models on 3 cancer type samples from Geneplus.

THCA						
	DeepTCR	DeepLION	MINN_SA	TransMIL	BiFormer	DeepLION2
ACC	0.733 ± 0.036	0.835 ± 0.039	0.740 ± 0.087	0.816 ± 0.043	0.840 ± 0.024	**0.886 ± 0.035**
SEN	0.481 ± 0.230	0.722 ± 0.101	0.542 ± 0.306	0.704 ± 0.111	0.729 ± 0.059	**0.751 ± 0.091**
SPE	0.891 ± 0.170	0.908 ± 0.016	0.874 ± 0.090	0.887 ± 0.052	0.911 ± 0.041	**0.973 ± 0.022**
MCC	0.457 ± 0.066	0.650 ± 0.087	0.447 ± 0.191	0.612 ± 0.091	0.662 ± 0.050	**0.765 ± 0.075**
AUC	0.860 ± 0.042	0.892 ± 0.043	0.843 ± 0.053	0.888 ± 0.036	0.917 ± 0.012	**0.933 ± 0.044**
LUCA						
	DeepTCR	DeepLION	MINN_SA	TransMIL	BiFormer	DeepLION2
ACC	0.721 ± 0.080	0.750 ± 0.072	0.655 ± 0.051	0.757 ± 0.063	0.768 ± 0.050	**0.809 ± 0.050**
SEN	0.393 ± 0.186	0.653 ± 0.109	0.620 ± 0.341	0.687 ± 0.123	0.716 ± 0.088	**0.736 ± 0.089**
SPE	**0.968 ± 0.044**	0.810 ± 0.082	0.711 ± 0.208	0.814 ± 0.070	0.812 ± 0.050	0.865 ± 0.037
MCC	0.463 ± 0.102	0.470 ± 0.158	0.358 ± 0.092	0.505 ± 0.114	0.525 ± 0.098	**0.606 ± 0.091**
AUC	0.836 ± 0.013	0.791 ± 0.075	0.788 ± 0.033	0.820 ± 0.067	0.836 ± 0.039	**0.880 ± 0.030**
GICA						
	DeepTCR	DeepLION	MINN_SA	TransMIL	BiFormer	DeepLION2
ACC	0.657 ± 0.034	0.650 ± 0.021	0.647 ± 0.033	0.681 ± 0.073	0.708 ± 0.032	**0.715 ± 0.061**
SEN	0.084 ± 0.093	0.292 ± 0.134	0.050 ± 0.076	0.286 ± 0.172	**0.470 ± 0.078**	0.288 ± 0.139
SPE	0.983 ± 0.027	0.865 ± 0.077	**0.993 ± 0.016**	0.922 ± 0.054	0.854 ± 0.092	0.970 ± 0.033
MCC	0.132 ± 0.137	0.193 ± 0.074	0.084 ± 0.141	0.243 ± 0.203	0.362 ± 0.090	**0.379 ± 0.074**
AUC	0.666 ± 0.085	0.617 ± 0.063	0.654 ± 0.067	0.704 ± 0.053	0.724 ± 0.024	**0.763 ± 0.032**

The maximum values of the evaluation metrics among the comparison models are shown in bold. THCA, thyroid cancer; LUCA, lung cancer; GICA: gastrointestinal cancer; ACC, accuracy; SEN, sensitivity; SPE, specificity; MCC, Matthews correlation coefficient; AUC, area under the receiver operating characteristic curve.

DeepLION2 showed superior performance compared to the other models in **Figure 3**, with higher average AUC validation results across the three cancer patient cohorts. The results in **Table 4** further confirmed DeepLION2’s consistent superiority, achieving impressive AUC values of 0.933, 0.880, and 0.763 for the THCA, LUCA, and GICA samples, respectively. Compared to DeepTCR, DeepLION and MINN_SA, TransMIL, BiFormer, and DeepLION2 exhibited better overall prediction performance by considering the correlations among TCRs in the repertoire. BiFormer, which addressed the sparsity of caTCRs and aimed to exclude unrelated TCRs, achieved higher ACCs, SENs, MCCs, and AUCs than TransMIL. However, its SPE performance on LUCA and GICA was weaker. To enhance attention learning, DeepLION2 employed self-contrastive learning during training, resulting in significant improvement in SPE metrics compared to BiFormer without compromising SEN metrics.

In comparison to the prediction performances on the preprocessed samples of the TCGA dataset (**Figure 2**, **Table 3** and **Supplementary Table 1**), DeepLION2 could produce more accurate predictions on these raw TCR-seq data because of the proper TCR antigen-specificity method. The AUC values of the predictions on three cohorts of the Geneplus dataset were all higher than the average AUC value of the predictions on the TCGA dataset (0.933, 0.880, and 0.763 for THCA, LUCA, and GICA > 0.735 for TCGA). Considering comparisons between samples of the same cancer type, the AUC value of the prediction on the LUCA cohort, consisting of LUAD and LUSC samples, was much higher than those on the LUAD and LUSC samples of the TCGA dataset (0.880 for LUCA > 0.639 and 0.498 for LUAD and LUSC). Although DeepLION2 performed exceptionally well on THCA samples, its performance was comparatively lower on the other two samples. This could be attributed to the inclusion of multiple cancer types or subtypes within the positive samples of LUCA and GICA, as well as the specificity of caTCRs for different cancer types/subtypes. Nevertheless, DeepLION2 consistently demonstrated high SPEs across all three cohorts, indicating its potential for cancer screening. Overall, DeepLION2 showcased a more accurate prediction of caTCRs and repertoires using TCR-seq data from patients with the same cancer type.

### 3.5 DeepLION2 unveils cancer-associated TCRs with key motifs for antigen-specific recognition in cancer repertoires

DeepLION2 could not only accurately predict the cancer status of patient samples but also identify caTCRs using TCR scores. Additionally, it could pinpoint key motifs of TCRs by calculating motif scores based on the weights of the trained model. In our experiments, we employed the trained models on test samples from THCA patient cohorts to reveal the associated cancer-specific TCRs along with their motifs.

**Figure 4:**
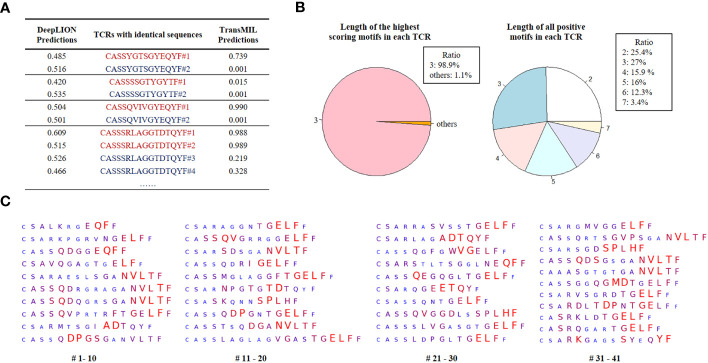
DeepLION2 unveils cancer-associated TCRs with key motifs for antigen-specific recognition in cancer repertoires based on the THCA patient cohorts. **(A)** The DeepLION and TransMIL predictions, the probability that a sequence is cancer-associated, on TCRs with identical sequences but from samples with different labels. Sequences from cancer repertoires are indicated by red, whereas those from non-cancer repertoires are indicated by blue. **(B)** The length distributions of the highest scoring motifs and all positive motifs (motif score > 0.5) in each TCR based on the predictions of DeepLION2. **(C)** DeepLION2 reveals top scoring TCRs (TCR score > 0.999, totally 41 sequences) and visualizes their key motifs. Larger, red-colored amino acids signify the model’s prediction of a more substantial role in antigen-binding.

Initially, we selected TCRs with identical CDR3 sequences but from samples with different labels to assess whether considering inter-TCR correlations could enhance the model’s performance when encountering these TCRs. The prediction results of DeepLION and TransMIL on these TCRs are shown in **Figure 4A**. DeepLION, without considering TCR correlations, yielded ambiguous predictions for these TCRs, hovering around 0.5. In contrast, when employing self-attention to account for inter-TCR correlations, TransMIL provided distinct predictions based on their contextual information. It is worth noting that TransMIL predicted low scores for TCRs with the CDR3 sequence “CASSSSGTYGYTF” from cancer and non-cancer samples, which suggested that within the cancer repertoire, this specific TCR might be considered as a background TCR unrelated to cancer.

Then, we analyzed the prediction results of DeepLION2 for TCRs with their motifs. We identified TCRs with scores above 0.5 within the cancer cohorts, indicating their potential likelihood of being caTCRs. The results indicated that the predominant length of the highest-scoring motif, most contributing to antigen-specificity within each of these TCRs, is 3 (**Figure 4B**). This finding is consistent with previous methods of preprocessing sequences into 3-length fragments to identify crucial motifs (10, 12). However, when considering all positive motifs detected by DeepLION2 (motif score > 0.5), their lengths ranged from 2 to 7, aligning with ratios observed in previous X-ray crystal structure analyses (**Figure 4B**) (14). Consequently, for TCR antigen-specificity extraction, it is essential to consider motifs of various lengths.

Ultimately, based on the scores of all positive motifs, we computed the amino acid weights of TCRs with a score > 0.999, which were highly probable caTCRs (**Figure 4C**). This analysis unveiled specific sequence segments DeepLION2 prioritized during predictions. Our assessment of 41 TCRs revealed the model’s consistent emphasis on the middle and rear sections of sequences, with less focus on the initial section containing similar amino acids like “CA” or “CS”. Moreover, it exhibited limited attention towards the final amino acid, “F,” except in certain specific combinations. Given our typical expectation of a greater emphasis on the middle sections of sequences due to their higher diversity, it’s intriguing that DeepLION2 directed its focus toward the rear sections of specific TCRs. While the diversity of amino acids in the CDR3 tail region is generally lower compared to those in the middle, and the rear sections of different TCRs might display higher similarity, certain scenarios suggest that amino acids in the rear sections could interact with specific parts of the antigenic peptide, potentially serving unique binding functions. On one hand, in many recent studies on TCR-peptide binding prediction, the prediction approaches have more or less reported a focus on amino acids in the rear sections of CDR3 sequences (16, 39, 40). On the other hand, some specific motifs in the rear sections were observed to appear more frequently in caTCRs compared to other cancer-unrelated TCRs, implying that we cannot ignore their important role in the cancer-associated antigen-binding process. For instance, the motif “NVLT”, frequently identified in the rear sections by DeepLION2 (presented in 9 out of 41 TCRs), appeared in 4.9% (145/2969) of caTCRs within the McPAS-TCR database, which is higher than the 2.1% (652/30714) occurrence observed in other TCRs (24). As a result, it’s logical for DeepLION2 to focus on the amino acids in the rear sections of CDR3 sequences, implying their potential significance in recognizing cancer-related antigens.

For further analysis of the identified TCRs and motifs by DeepLION2, we cross-referenced them with the CEDAR and McPAS-TCR databases, renowned for their collection of known caTCRs (24, 41). We first searched for the 41 TCRs in two databases, but we didn’t find identical sequences in either caTCRs or TCRs unrelated to cancer, which may be due to the high diversity of TCRs. Meanwhile, because different types of cancer are highly heterogeneous, it is reasonable that these 41 TCRs specific to THCA were not present in these databases for cancer, containing few TCRs for THCA. Next, upon investigating the motifs that DeepLION2 highlighted in the databases, we observed that certain motifs revealed by DeepLION2 appeared in caTCRs in both two databases. And we also observed that some motifs occurred more frequently in caTCRs compared to other TCRs, such as “NVLT” as previously mentioned. Some motifs, such as “QDPGS” and “QDPGN” (in #10 and 18 TCRs), were even exclusive to caTCRs in the McPAS-TCR database, indicating their potential as THCA-specific biomarkers and promising targets for cancer immunotherapy. Furthermore, the model’s preference for non-adjacent amino acids in most TCRs could be attributed to the structural folding of proteins, where amino acids binding to antigen peptides are not sequentially adjacent.

### 3.6 Impact of hyperparameters on DeepLION2 prediction performance

**Figure 5:**
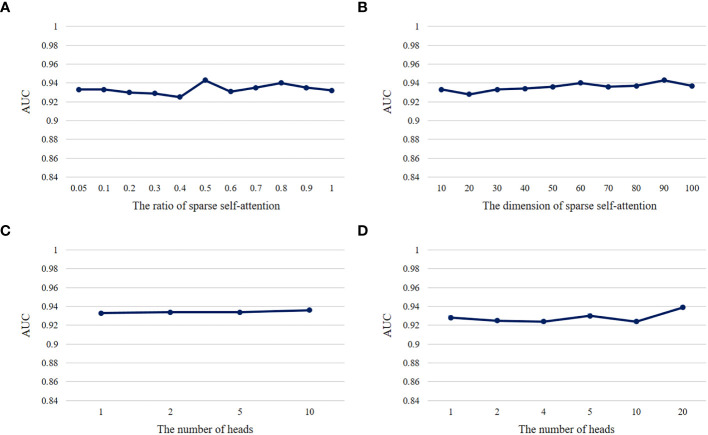
The AUC results of models with different hyperparameters on THCA samples. **(A)** The AUC results of the DeepLION2 models with different ratios of sparse self-attention. **(B)** The AUC results of the DeepLION2 models with different dimensions of self-attention. **(C)** The AUC results of the DeepLION2 models with the dimension of self-attention as 10 and different head numbers of self-attention. **(D)** The AUC results of the DeepLION2 models with the dimension of self-attention as 20 and different head numbers of self-attention.

Hyperparameters play a crucial role in the performance of a model. We conducted ablation experiments about the important hyperparameters D' , *h*, *k_r_* in DeepLION2 to validate their influence on model performance. In each group of ablation experiments, we changed only the hyperparameters to be observed while keeping the other hyperparameters unchanged and employed 5-4-fold nested cross-validation to test the models on the THCA patient cohorts. The metric AUC was used to evaluate the models and the validation results are shown in **Figure 5**. According to the results, we observed that the model performance was overall stable and unaffected by these hyperparameter changes. It is worth noting that multi-head self-attention did not achieve a higher accuracy than one-head self-attention in caTCR prediction, which may be due to the small sample size of TCR-seq data. Among the other hyperparameters, *N* was discussed in DeepLION (17) and set to 100 for the tradeoff between model performance and computational cost. *l_r_* was usually set to 0.001, whereas due to the use of validation sets and the early stopping approach, *e* would not affect the model performance as long as the model converged during the training process.

## 4 Discussion

In this study, we developed a novel deep MIL learning method, named DeepLION2, for improving the prediction of caTCRs and repertoires, which incorporated content-based sparse attention and contrastive learning in its MIL part. Compared to the existing methods, it used sparse self-attention to fully consider the correlations among TCRs and avoided incorrectly predicting TCRs with the same/similar CDR3 sequences as caTCRs. Furthermore, to ensure that the model correctly focused on caTCRs, it used the self-contrastive learning mechanism to improve attention learning. To validate the improvement of DeepLION2, we collected patient samples of more than ten cancer types from TCGA and Geneplus. The results indicated that DeepLION2 generally outperformed the comparison models across the preprocessed ten cancer samples from TCGA. Moreover, the results on the raw TCR-seq data of three cancer patient cohorts from Geneplus also highlighted that DeepLION2 could advance the prediction of caTCRs and repertoires, where its AUC values reached notably 0.933, 0.880, and 0.763 on the THCA, LUCA and GICA patient cohorts, respectively.

To mitigate overfitting concerns, we took several steps in our experiments. Firstly, we simplified the model structure by using only one-layer self-attention/sparse self-attention, which helps prevent overfitting when the training data is limited. Additionally, we incorporated random dropout with a rate of 40% during training, a well-established technique known for effectively reducing overfitting and widely used in various machine learning models (42). Furthermore, we employed the early-stopping approach to prevent the model from overtraining. By monitoring the model’s performance on validation sets, we stopped the training process at an appropriate time to avoid performance degradation on the test sets (43). This approach helps ensure that the model generalizes well to unseen data. Moreover, the utilization of nested cross-validation, a robust and unbiased validation technique, further reinforced the outstanding performance of DeepLION2 in predicting caTCRs. By validating the model on multiple folds of the data, we obtained reliable and comprehensive performance estimates, enhancing the confidence in the model’s predictive capabilities.

In the comparison experiments conducted on both the TCGA and Geneplus datasets, DeepLION2 consistently outperformed existing methods. This can be attributed to its utilization of content-based sparse self-attention to effectively model the correlations among TCRs, along with the incorporation of self-contrastive learning to enhance attention learning. Notably, as described in Section 3.4, the performance of the models on the preprocessed samples from the TCGA dataset was inferior to that on the raw TCR-seq samples from the Geneplus dataset. This discrepancy can be attributed to the differences in the approaches used for TCR antigen-specificity extraction between the two datasets. In the TCGA dataset, stacked auto-encoders were employed for TCR feature extraction. However, this approach did not take into account the key motifs of different lengths present in the TCR CDR3 sequences. On the other hand, the raw TCR-seq samples from the Geneplus dataset were processed using a convolutional network with filters of different sizes, allowing for the handling of fragments with varying lengths in TCRs. Hence, the methodology used for TCR antigen-specificity extraction plays a crucial role in predicting caTCRs, and it is this aspect that contributes to the outstanding performance of DeepLION2.

While proficiently discerning cancer-associated patient repertoires, DeepLION2 concurrently identifies caTCRs within these repertoires, shedding light on key motifs. The model’s emphasis on the rear sections of CDR3 sequences from the 41 TCRs in THCA patient cohorts aligns with previous research more or less focusing on the amino acids in such sections. Notably, certain motifs occurring more frequently in caTCRs compared to non-cancer-related TCRs underscore the significance of DeepLION2’s attention to these rear-section amino acids. It is crucial not to overlook these amino acids when studying TCR-peptide binding. It’s worth noting that DeepLION2’s focus on the 41 TCRs and their motifs does not necessarily imply their direct association with cancer or involvement in binding to cancerous antigens. The attention mechanism indicates the features contributing to the classification between cancerous and non-cancerous repertoires, suggesting potential caTCRs and amino acids relevant to cancer antigen recognition and binding. For a deeper analysis, we cross-referenced these results with existing cancer databases. Due to the vast diversity of TCRs and the heterogeneity of cancers, the 41 TCRs from THCA did not appear in the caTCR or non-cancer-related TCR lists in the databases. Nevertheless, certain motifs identified by DeepLION2 were found in caTCRs in both databases. Additionally, some motifs were more prevalent in caTCRs, with a few exclusive to caTCRs. These findings hint at the potential of these motifs as THCA-specific biomarkers, supporting the validity of slicing TCRs into motifs for consideration.

In future work, we aim to further validate the performance of DeepLION2 by applying it to a broader range of cancer types. We acknowledge that the performance of DeepLION2 experienced a decline when samples contained multiple cancer types and when the size of the training samples was smaller. To address this, we plan to enhance the model to more effectively extract the specificity of caTCRs from limited data, thereby improving its performance in such scenarios. Furthermore, we recognize that the presence of noise in TCR-seq data poses a limitation on the model’s performance. This is an important issue that we intend to address in future research. By developing techniques to mitigate the impact of noise in TCR-seq data, we aim to enhance the robustness and accuracy of DeepLION2 for predicting caTCRs and advancing its practical utility in clinical settings. In addition, it has been recognized that the α chain, a constituent of the TCR along with the β chain, also plays a significant role in the recognition of antigens. For a more comprehensive understanding of the antigen recognition mechanism of the TCR, we will further consider the α chain and develop models to support the analysis of both chains.

## 5 Conclusion

DeepLION2 is a groundbreaking deep MIL framework that integrates content-based sparse attention and contrastive learning to capture TCR correlations in a repertoire. It outperforms existing methods in accurate caTCR and repertoire prediction from TCR-seq data. Additionally, it can unveil potential caTCRs and their crucial motifs. DeepLION2 enables effective repertoire classification, potentially supporting cancer detection and facilitating personalized cancer immunotherapy.

## Data availability statement

DeepLION2 is available on GitHub, at https://github.com/Bioinformatics7181/DeepLION2, for academic use only. The preprocessed samples of the TCGA dataset were from Xiong’s study (33), which can be found at https://github.com/danyixiong/MIL_Comparative_Study. In the context of the Geneplus dataset, the THCA TCR-seq samples were from Lan’s study (34), which can be found in NCBI, at https://www.ncbi.nlm.nih.gov/bioproject/PRJNA642967, whereas the LUCA samples were from Li’s study (36) and the GICA samples were from Ji’s study (35). All the processed data used in the experiments can be found at https://github.com/Bioinformatics7181/DeepLION2.

## Ethics statement

Ethical approval was not required for the studies on humans in accordance with the local legislation and institutional requirements because only commercially available established cell lines were used.

## Author contributions

XQ: Data curation, Methodology, Validation, Visualization, Writing – original draft, Writing – review & editing. GY: Data curation, Investigation, Methodology, Validation, Writing – review & editing. FL: Writing – review & editing. XuZ: Supervision, Writing – review & editing. XiZ: Writing – review & editing. XL: Writing – review & editing. XX: Writing – review & editing. TW: Supervision, Writing – review & editing. JW: Funding acquisition, Supervision, Writing – review & editing.
